# Neuroprotective Effects and Mechanisms of Carvacrol and Thymol in Alzheimer’s Disease: A Scoping Review

**DOI:** 10.3390/ph19091407

**Published:** 2026-09-06

**Authors:** Shabbir Adnan Shakir, Juen Kiem Tan, Kok-Yong Chin

**Affiliations:** 1 Department of Pharmacology, Faculty of Medicine, Universiti Kebangsaan Malaysia, Cheras 56000, Malaysia; 2 Department of Medicine, Faculty of Medicine, Universiti Kebangsaan Malaysia, Cheras 56000, Malaysia

**Keywords:** cognitive impairment, dementia, monoterpenes, neuroinflammation, oxidative stress

## Abstract

**Background/Objectives:** Alzheimer’s disease (AD) is a multifactorial neurodegenerative condition characterised by cognitive impairment, cholinergic deficiency and neuroinflammation. Carvacrol and thymol are two related natural monoterpenes with reported antioxidant, anti-inflammatory, anti-apoptotic and cholinesterase-inhibiting properties. This scoping review aims to map the current evidence regarding the effects of carvacrol and thymol on AD-related pathologies and identify their mechanisms of action. **Methods:** A systematic literature search was conducted in February 2026 across PubMed, Scopus and Web of Science. This review included original, English-language primary research articles investigating the effects of carvacrol and thymol on AD using in vitro, in vivo, or in silico models. **Results:** From an initial pool of 87 unique records, 30 primary studies met the inclusion criteria, encompassing direct AD pathology models (e.g., amyloid-beta), AD-associated risk models (e.g., metabolic or hypertensive impairment), and general cognitive impairment models. Both carvacrol and thymol reduced escape latency during spatial learning acquisition training and increased time spent in the target quadrant during probe trials, indicating improvements in both learning and memory retention, although several studies reported non-linear relationships. At the cellular level, carvacrol and thymol modulated distinct redox and inflammatory pathways, including nuclear factor erythroid 2-related factor 2 upregulation and tumour necrosis factor-alpha suppression. Synthetic derivatives and nanocarrier formulations (e.g., liposomes, nanoemulsions) demonstrated enhanced acetylcholinesterase inhibition and biological stability relative to parent monoterpenes. No clinical trials were identified, and formal critical appraisal was not performed. **Conclusions:** Preclinical evidence indicates that carvacrol and thymol show potential multi-targeted neuroprotective activity across diverse models of cognitive dysfunction. However, clinical translation remains unproven due to heterogeneous experimental designs, pharmacokinetic limitations, a lack of human trials, and unassessed study quality.

## 1. Introduction

Alzheimer’s disease (AD) is a debilitating neurodegenerative disorder and the leading cause of dementia among the elderly. It is characterised by progressive cognitive decline, memory impairment and profound personality changes. As the global population continues to age, the prevalence of AD is projected to rise significantly, which will pose an immense socioeconomic burden on healthcare systems and caregivers worldwide [1,2]. Globally, dementia prevalence was estimated at 57.4 million in 2019 and is projected to increase to 152.8 million by 2050, with women affected more often than men [3]. These trends highlight the scale of the problem and reinforce the need for interventions that go beyond symptomatic relief. Among other factors, insulin resistance, metabolic syndrome, menopause and chronic cerebral hypoperfusion are recognised as risk factors for AD [4,5,6].

The classic amyloid cascade hypothesis suggests that AD is caused by extracellular accumulation of amyloid-beta (Aβ) peptides into insoluble plaques, leading to synaptic dysfunction and eventual neuronal death [7,8,9]. Modern research recognises that the aetiology of AD is multifactorial and complex, involving several interlocking pathological pathways. These pathways include Aβ accumulation, tau hyperphosphorylation, synaptic dysfunction, oxidative stress, and neuroinflammation [10].

Approved pharmacological treatment for AD includes symptomatic therapy and disease-modifying agents. As these agents are less accessible, management primarily involves the use of acetylcholinesterase inhibitors (AChEIs), such as donepezil and rivastigmine, as well as N-methyl-D-aspartate (NMDA) receptor antagonists like memantine [11,12,13,14]. These agents are effective in relieving AD’s symptoms, but do not adequately modify the underlying pathological cascade [15,16]. This therapeutic gap has encouraged interest in natural compounds with pleiotropic actions, especially those capable of targeting oxidative stress, inflammation and neurotransmitter dysfunction simultaneously [15,16].

Carvacrol and thymol are phenolic monoterpenes commonly found in essential oils from *Lamiaceae* plants such as thyme and oregano [17,18]. Carvacrol (5-isopropyl-2-methylphenol) and thymol (2-isopropyl-5-methylphenol) are positional isomers, sharing the same molecular formula (C_10_H_14_O) but differing in the placement of the hydroxyl and isopropyl groups on the aromatic ring. Monoterpenes have attracted considerable attention for their ability to cross the blood–brain barrier (BBB) and exert neuroprotective effects [17]. Both compounds have been associated with antioxidant and anti-inflammatory activity, which is especially relevant in AD because oxidative injury and inflammatory signalling are involved in neuronal loss [17,18,19]. Moreover, these compounds have been traditionally used in folk medicine for their antimicrobial and antiseptic properties, but recent studies have highlighted their ability to modulate various pathways involved in neurodegeneration [17,18,20,21].

Taken together, the available evidence suggests that carvacrol and thymol may act as multi-target neuroprotective agents in AD by combining antioxidant, anti-inflammatory, anti-cholinesterase, and cell-survival effects. Despite accumulating evidence from individual studies, the literature on the specific effects of carvacrol and thymol in AD models remains fragmented across experimental designs and dosages. While existing reviews have broadly discussed the therapeutic potential of plant-derived monoterpenes [22,23,24], significant knowledge gaps remain regarding the specific mechanisms and comparative profiles of carvacrol and thymol in AD. Available primary studies exhibit considerable heterogeneity in disease models, dosage regimens, and formulations. This scoping review aims to address these gaps by systematically mapping preclinical evidence, clarifying compound-specific mechanistic pathways, and identifying crucial translational barriers to guide future research.

## 2. Results

**Figure 1:**
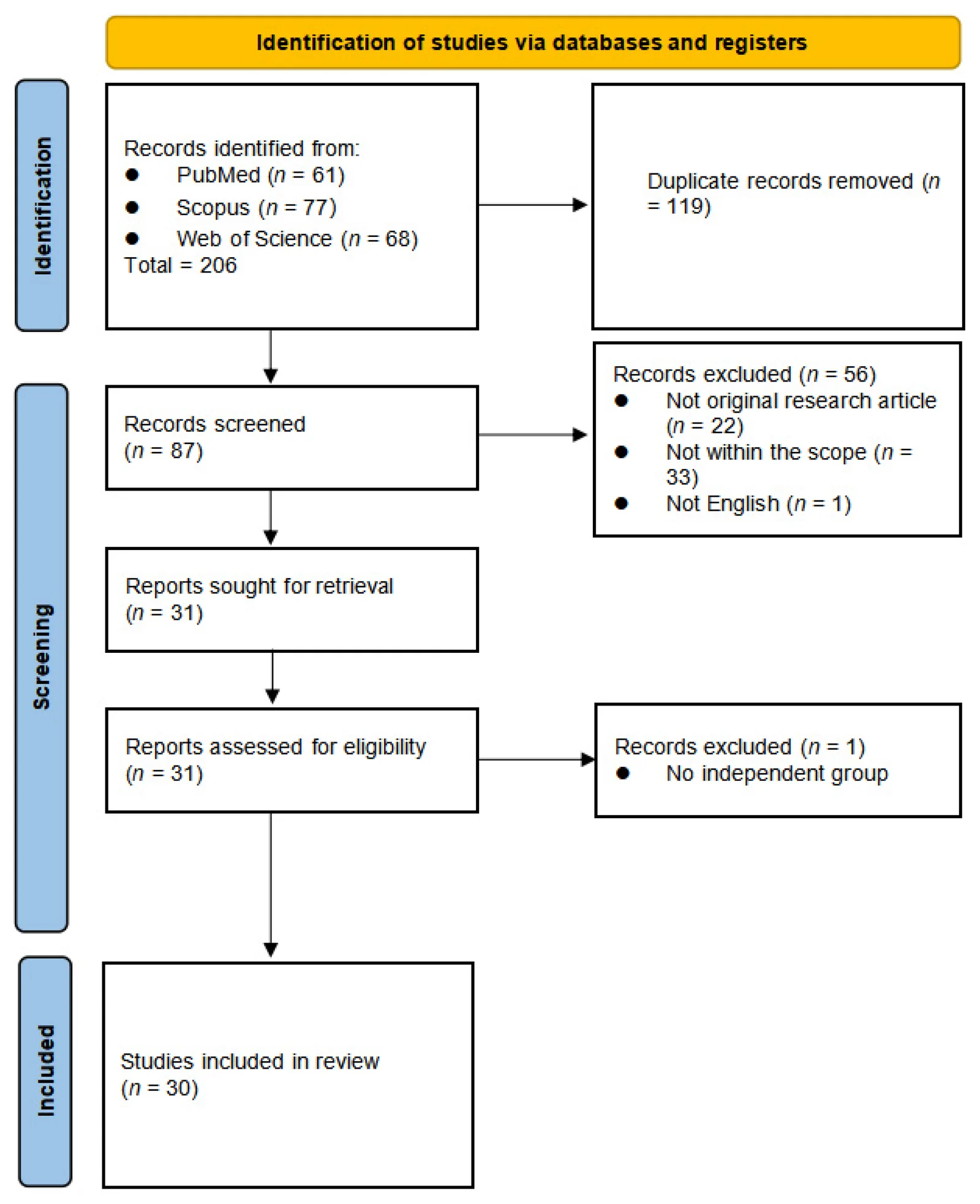
PRISMA flowchart on the article selection process.

The database search identified 87 unique records, of which 30 primary studies met the final inclusion criteria (Figure 1). These included 15 in vivo-only studies, 4 in vitro-only studies, 2 studies that used both in vivo and in vitro methods, 1 study that used both in vivo and in silico methods, 6 studies that used both in vitro and in silico methods, 1 study that used all three methods (in vivo, in vitro, and in silico), as well as 1 in silico-only study. Cumulatively, this translates to 19 studies with an in vivo component, 13 with an in vitro component, and 9 with an in silico component. No clinical trial evaluating carvacrol or thymol in AD was identified. Publication years of the articles ranged from 2011 to 2026.

### 2.1. Study Characteristics

#### 2.1.1. AD Models

The models used to evaluate the effects of monoterpenes can be broadly divided into direct AD models, associated AD-risk factor models, and general cognitive impairment models. The direct AD models are restricted to models simulating hallmark neuropathologies, particularly Aβ microinjections, formaldehyde-induced tau phosphorylation, or genetic risk factors. In these settings, interventions target physical peptide aggregation and the destruction of synaptic plasticity. In vivo, bilateral intrahippocampal microinjections of Aβ_25–35_ in rats cause localised cellular necrosis in the CA1 pyramid, suppress long-term potentiation (LTP), and impair spatial memory [25,26]. In vitro, Aβ_25–35_ triggers rapid generation of reactive oxygen species, caspase-3 cleavage, and metabolic death in PC12 and SH-SY5Y cells [27,28]. Beyond Aβ, tau-relevant mechanisms are modelled via formaldehyde-induced tau hyperphosphorylation and aggregation in PC12 cells [29], while the pathology of genetic AD is modelled using transgenic models or ApoE4-induced neurotoxicity, which downregulates the vital reelin/LRP8 signalling pathway required for synaptic maturation [30].

The associated AD-risk models represent chronic systemic conditions that clinically predispose patients to vascular dementia and accelerate AD onset, rather than simulating primary AD pathology itself. Metabolic models utilise high-fat diet (HFD) regimens in rodents to induce peripheral obesity and hyperlipidaemia, leading to localised brain insulin resistance via inhibitory serine phosphorylation of insulin receptor substrate-1 (IRS-1), thereby impairing hippocampal AKT/glycogen synthase kinase (GSK)-3β signalling [31]. Endocrine-metabolic risk is simulated using streptozotocin (STZ) to destroy pancreatic beta cells, resulting in type 1 diabetes-associated cognitive deficit (DACD) driven by chronic hyperglycaemia [32]. Vascular risk is modelled via chronic cerebral hypoperfusion, specifically bilateral common carotid artery occlusion or the 2-vessel occlusion (2VO) model, which induces severe ischaemia and ischaemic pyramidal death [33]. Finally, combined vascular-endocrine risks are evaluated in ovariectomised renovascular hypertensive (OVX/RVH) models, which simulate oestrogen-depleted postmenopausal hypertension [34]. Results in these frameworks indicate broader, systemic glucose-lowering, lipid-modulating, and vaso-protective activities rather than AD-specific peptide clearance.

The general cognitive impairment models evaluate generalised neuroprotection, transient amnesia, or neuroinflammation without replicating AD-specific plaques or neurofibrillary tangles. Scopolamine-induced amnesia is widely utilised as a chemical model of acute, transient cholinergic hypofunction by reversibly blocking muscarinic receptors [35]. Acute neuroinflammation is modelled through systemic injections of bacterial lipopolysaccharide (LPS) to prompt microglia activation and a massive surge in pro-inflammatory cytokines [36]. Chronic stress models, such as chronic unpredictable mild stress (CUMS), simulate clinical depressive states and working memory fatigue without primary physical cell death [37]. These models demonstrate general adaptogenic, anti-inflammatory, or anticholinesterase activities that broadly modulate cognition.

#### 2.1.2. Interventions

In studies using the native monoterpenes, acute, low-dose thresholds were demonstrated in vivo (such as thymol at 0.5 mg/kg and carvacrol at 1 mg/kg) for transient memory rescue [25] and in vitro neuroprotection at 10–50 μM [27], but high-dose chronic administration was required (25–100 mg/kg) to suppress systemic, chronic metabolic indices [31,32].

Advanced delivery systems alter the pharmacokinetics and therapeutic thresholds of the parent compounds. A 24 nm spherical carvacrol oil-in-water nanoemulsion stabilised with Tween 80 demonstrated significantly enhanced thermodynamic stability and BBB permeation, dramatically outperforming raw carvacrol oil in restoring central monoamines and suppressing urinary 8-OHdG and advanced oxidation protein products [38]. Similarly, soy phosphatidylcholine liposome-encapsulated thymol (LET) protects thymol from rapid in vivo clearance and oxidation, yielding a twofold greater recovery of hippocampal brain-derived neurotrophic factor (BDNF) and significantly higher step-through latencies compared to equivalent doses of free thymol [39].

Chemical modifications of the monoterpene scaffold are designed to drastically enhance their low native anticholinesterase potency, and their results cannot be extrapolated to the parent compounds. Triazole-linked organoselenium carvacrol hybrids (compounds **18e** and **18h**) achieve exceptionally potent, nanomolar-range AChE inhibition (IC_50_ of 75 nM and 93 nM, respectively), representing a several thousand-fold increase over native carvacrol [40]. Carbamate substitutions on carvacrol and thymol yield up to a 20,500-fold increase in butyrylcholinesterase (BuChE) inhibition (compound **30**, IC_50_ of 0.02 μM) and up to a 130-fold increase in AChE inhibition (compound **29**, IC_50_ of 2.22 μM) [41]. Additionally, novel carvacrol-substituted amides (compound **5v**) achieve dual AChE/BuChE inhibition at 1.93 μM and 0.05 μM [42], and carvacrol-derived sulphonamides act as potent cognitive enhancers while lowering brain lipid peroxidation in vivo [43].

Studies investigating complex mixtures contain compounding synergistic or antagonistic volatile constituents. Co-administration can lead to unexpected drug–drug interactions. Chronic co-administration of carvacrol (50 mg/kg) and p-cymene (50 mg/kg) completely abolishes the hippocampal LTP rescue achieved by either compound individually, indicating severe functional antagonism or metabolic competition when these monoterpenes are combined [26].

### 2.2. Neurological Outcomes

#### 2.2.1. Effects of Carvacrol

Carvacrol demonstrates neuroprotective effects across multiple experimental models, primarily by preserving cognitive function and synaptic plasticity. During acquisition training, carvacrol consistently reduced escape latency and travel distance across diverse pathological states, including STZ-induced diabetes [32], chronic cerebral hypoperfusion [33], OVX/RVH [34], LPS-induced neuroinflammation [36] and CUMS [37]. Similarly, in probe trials, treated animals spent significantly more time in the target quadrant compared to negative controls, indicating improved spatial memory retention. Electrophysiologically, it protects perforant path–dentate gyrus (PP-DG) LTP induction against Aβ-induced deficits [26]. At the cellular level, in vitro evidence shows that carvacrol shields SH-SY5Y cells from Aβ toxicity by preserving normal morphology, restoring mitochondrial membrane potential, and downregulating cleaved caspase-3 activation [28].

Carvacrol also exerts distinct systemic vascular and metabolic actions. Systemically, it lowers plasma glucose and attenuates diabetic weight loss in STZ-injected rats [32], while also reducing systolic blood pressure independently of oestrogen status in OVX/RVH models [34]. Biologically, these physiological outcomes are supported by a universal suppression of (tumour necrosis factor) TNF-α, (interleukin) IL-1β and (nuclear factor-kappa B) NF-κB, accompanied by reduced lipid peroxidation markers [MDA (malondialdehyde) and (thiobarbituric acid reactive substances) TBARS] and the restoration of endogenous antioxidant enzymes such as superoxide dismutase (SOD) and catalase (CAT) [32,33,36] (Table 1).

#### 2.2.2. Thymol’s Effects

In vivo investigations show that thymol reverses cognitive impairment in scopolamine-induced amnesia [35], lead neurotoxicity [45], high-fat diet (HFD) dysfunction [46], and ApoE4-mediated neurodegeneration [30]. Electrophysiologically, it rescues both the slope and population spike amplitude of PP-DG LTP under HFD and Aβ_1–42_ stress [46]. At the molecular level, in vitro studies demonstrate that thymol reverses formaldehyde-induced tau hyperphosphorylation and aggregation in PC12 cells by upregulating protein phosphatase 2A (PP2A) expression [29].

Systemically, thymol regulates lipid profiles and peripheral insulin sensitivity, while selectively engaging central neurotrophic pathways. Daily treatment (30 mg/kg) in HFD-fed rats corrects hyperlipidaemia by lowering triglycerides, VLDL, and total cholesterol without altering overall body weight or blood glucose [46]. Conversely, chronic administration at higher doses (20 and 40 mg/kg) in obese mice successfully reverses body weight gain and peripheral HOMA insulin resistance. This peripheral metabolic gain is driven centrally by the downregulation of inhibitory brain p-Ser307 IRS-1 and the upregulation of downstream p-AKT and p-GSK-3β signalling [31]. Biochemically, thymol stimulates adult hippocampal neurogenesis (increasing doublecortin-positive cells), rescues hippocampal BDNF levels [35]. Another study reported that thymol improved recognition and spatial memory in the D-galactose/AlCl_3_ model and significantly reduced brain ApoE levels, although it did not significantly restore reelin or LRP8 levels compared with the disease group [30] (Table 2).

#### 2.2.3. Comparative Evidence Between Thymol and Carvacrol

Direct side-by-side evaluations highlight a fundamental paradox between in vitro cholinesterase inhibition and in vivo cognitive efficacy for these two isomers. In cell-free assays, carvacrol is a much more potent AChE inhibitor than thymol, with an IC_50_ of 3.8–64.21 µg/mL compared to thymol’s IC_50_ of 565.7 µg/mL [51]. Structural modelling attributes this difference to steric factors, whereby carvacrol’s para-hydroxyl group fits tightly into the active site cavity of human AChE, forming direct hydrogen bonds with Asp74 and Tyr341 to yield a lower binding free energy than thymol’s meta-hydroxyl configuration [51]. However, in vivo behavioural studies show that thymol performs as well as or better than carvacrol in reversing Aβ- and scopolamine-induced amnesia in rodents [25]. This divergence suggests that in vitro cholinesterase potency does not directly predict behavioural recovery, implying that thymol possesses superior central bioavailability or blood–brain barrier transport kinetics.

Comparative cellular assays further delineate the distinct safety margins and primary mechanistic targets of each compound. In PC12 cell models, both isomers (10–50 μM) protect against Aβ_25–35_-induced toxicity and ROS by activating the PKC pathway, particularly by upregulating PKCα expression [27,52]. However, safety profiles on healthy baseline cells favour carvacrol. Thymol triggers significant cytotoxicity at concentrations exceeding 100 μM, whereas carvacrol remains non-toxic up to 200 μM [27]. Conversely, cell-free aggregation assays reveal that thymol is a significantly more potent inhibitor of direct Aβ-oligomerization and fibrillization than carvacrol [51]. Taken together, these comparative insights suggest that carvacrol primarily acts as a safer modulator of cellular and synaptic AChE, while thymol preferentially acts as a direct peptide anti-aggregation agent (Table 3).

## 3. Discussion

Across the diverse experimental designs, carvacrol and thymol supplementations show a potential therapeutic profile in mitigating central nervous system deficits. Behavioural assessments revealed consistent improvements across both phases of the Morris Water Maze. Escape latency and travel distance decreased during spatial learning acquisition, while time in the target quadrant and platform crossings increased during probe trials. These changes collectively indicate enhanced learning and memory retention [25,28,32,35,39]. These cognitive improvements correspond to several consistent molecular and cellular mechanisms of action, which have been reported across independent studies.

Firstly, both compounds suppress pro-inflammatory cascades, as evidenced by a downregulation of TNF-α, IL-1β, IL-6, NF-κB p65 subunit and COX-2 in the circulation and hippocampus [32,36,37,39]. Secondly, both compounds attenuate oxidative stress and brain tissue damage by decreasing lipid peroxidation and advanced oxidation protein products, while restoring endogenous antioxidant enzymatic and non-enzymatic systems [28,32,37,47]. Thirdly, at the synaptic level, carvacrol and thymol preserve central cholinergic transmission. They reverse AChE hyperactivation and upregulate BDNF transcription pathways [35,39]. Biochemically, both compounds counteract Aβ neurotoxicity and apoptosis by inhibiting caspase-3 cleavage and directly upregulating the PKC pathway, specifically upregulating PKCα expression, which is associated with the prevention of necrosis and nuclear shrinkage of CA1 pyramidal neurons [27,28,32,52].

At the molecular level, independent groups have replicated the upregulation of the protective Nrf2/HO-1 pathway by thymol or carvacrol in HFD-induced cognitive decline, lead-induced neurotoxicity, and LPS-induced neuroinflammation [31,36]. Furthermore, the inhibition of caspase-3 apoptotic cascades and the correction of GSK-3β phosphorylation (Ser9 inactivation) have been reported across scopolamine amnesia, aluminium chloride (AlCl_3_) metal toxicity, and HFD-induced cognitive deficits [31,35,45]. Most recently, Bitmez et al. (2024) confirmed that thymol downregulates ApoE4-mediated neurotoxicity, but the study did not provide sufficient evidence for thymol’s effects in restoring the reelin/LRP8 signalling pathway [30].

Despite the consistency of their therapeutic effects, some controversies and non-linear biological responses have been noted. The first controversy is about the effects of co-administration of carvacrol and p-cymene on long-term potentiation [26]. In an in vivo electrophysiological study of PP-DG synapses, chronic treatment with carvacrol (50 mg/kg, gavage) or its monoterpene precursor p-cymene (50 mg/kg) individually prevented Aβ_1–42_-induced deficits in population spike (PS) amplitude and fEPSP slope. However, co-administration of carvacrol and p-cymene failed to confer any neuroprotective effect, leaving long-term potentiation induction severely compromised. The authors proposed antioxidant stress as an explanation for this antagonism, in which normal physiological levels of ROS act as necessary signalling molecules to induce synaptic plasticity. However, excessive radical-scavenging activity by both antioxidants ameliorates ROS needed for physiological processes, thereby disrupting the redox signalling required for normal long-term potentiation induction [26].

The second controversy concerns the contradictory results between in vitro and in vivo findings. Enzymatic assays confirm that carvacrol is a substantially stronger AChE inhibitor than thymol in vitro [51]. Molecular modelling suggests this difference is steric because carvacrol’s para-hydroxyl group allows a tighter configuration and stronger binding free energy in the beta-secretase and AChE binding cavities compared to thymol’s meta-hydroxyl structure [51,54]. However, in vivo cognitive testing in rodents reveals that thymol is at least as effective as carvacrol in reversing Aβ- and scopolamine-induced amnesia [25]. This discrepancy underscores that in vitro AChE potency does not directly predict in vivo cognitive restoration, indicating that in vivo absorption, BBB penetration kinetics, and active metabolite generation are more favourable for thymol.

Carvacrol and thymol have been evaluated across several distinct AD models, suggesting that their therapeutic profiles vary depending on the underlying disease aetiology. In diabetic rats, chronic treatment with carvacrol (25, 50, and 100 mg/kg for 7 weeks) remarkably attenuated DACD [32]. Strikingly, carvacrol also exerted significant systemic metabolic improvements, lowering blood glucose levels and preventing severe diabetic-related weight loss. This demonstrates that in type 1 diabetes mellitus, carvacrol acts as a dual peripheral hypoglycaemic and central neuroprotective agent, likely by attenuating systemic pancreatic inflammation and caspase-3-mediated apoptotic cascades in brain tissue. In contrast, the metabolic outcomes of thymol in HFD models depend heavily on species and duration. In Wistar rats fed an HFD for 8 weeks and then injected with Aβ_1–42_, oral thymol (30 mg/kg) daily for 4 weeks corrected dyslipidaemia but did not alter overall body weight or non-fasting blood glucose [46]. Conversely, in obese C57BL/6J mice fed an HFD for 12 weeks, daily oral thymol (20 and 40 mg/kg) successfully reversed both body weight gain and peripheral insulin resistance [31]. On a molecular level, thymol appears to resolve hippocampal insulin resistance by downregulating the inhibitory phosphorylation of insulin receptor substrate-1 at serine 307 (P-Ser307 IRS-1), thereby restoring the vital downstream P-Ser473 AKT and P-Ser9 GSK-3β signalling cascade. In rats with postmenopausal hypertension, carvacrol (40 mg/kg, p.o.) significantly reduced systolic blood pressure in an oestrogen-independent manner [34]. It also lowered cortical and hippocampal Aβ deposition and decreased osteopontin and pro-inflammatory cytokines, demonstrating its efficacy under combined vascular and endocrine stressors. In rats with bilateral carotid artery occlusion (2VO model), carvacrol (25 and 50 mg/kg) directly protected the highly hypoxia-sensitive CA1 hippocampal pyramidal cells from necrotic cell death and nuclear shrinkage, primarily by suppressing brain MDA levels and increasing catalase and SOD activities [33].

The literature establishes a clear distinction between the dosing windows required for acute memory rescue and those for chronic metabolic and anti-inflammatory therapies. For acute pretraining memory rescue, extremely low intraperitoneal doses of thymol (0.5 mg/kg) and carvacrol (1.0 mg/kg) are highly effective in reversing scopolamine- and Aβ-induced amnesia [25]. However, in systemic metabolic, diabetic, and chronic stress models, much higher, chronic daily doses (25, 50, and 100 mg/kg) are necessary to produce measurable reductions in peripheral and central inflammatory markers, Aβ plaques, and glycaemic parameters [32,37,46]. In an LPS-induced neuroinflammation model, carvacrol at 25 mg/kg significantly resolved cognitive impairments, but doubling the dose to 50 mg/kg did not confer additional therapeutic benefit [36]. These therapeutic ranges are well below the rodent lethal doses (the intraperitoneal LD_50_ values are 565.7 mg/kg for thymol and 471.2 mg/kg for carvacrol) [25], confirming a wide therapeutic index. In PC12 cells, both compounds are highly neuroprotective against Aβ25–35-induced cell death and ROS generation at concentrations of 10 to 50 μM by upregulating PKC activity [27]. However, healthy PC12 cells exhibit significant cytotoxicity at higher doses. Cell viability declines if thymol exceeds 100 μM or if carvacrol exceeds 500 μM [27]. Similarly, in SH-SY5Y cells, both compounds are safe and protective at 5–50 μg/mL, but healthy cells exhibit a significant reduction in metabolic viability at concentrations of 100 μg/mL and above [44,51].

In terms of pharmacokinetics, thymol and carvacrol display rapid absorption from the upper gastrointestinal tract but limited systemic availability of the free parent compounds, with extensive first-pass and phase II metabolism. A single-dose (1.08 mg thymol) study in humans reported that free thymol was not detectable in plasma after oral administration. Systemic exposure was observed only as thymol sulphate, with around 16.2% of the dose recovered as phase II metabolites in 24 h urine [55]. Comparative animal data show near-complete early absorption but substantial distal-gut losses and tissue sequestration. A study in piglets showed that carvacrol and thymol peak in the plasma at 1.4 and 1.35 h, respectively, with digestive-tract half-lives of 1.8–2.0 h. They were mainly absorbed in the stomach and proximal small intestine, with detectable degradation in caecal simulations up to 29% [56]. A study in rabbits documented much higher thymol concentrations in the intestinal wall than in plasma or liver. The researchers correlated plasma–liver relationships with intensive hepatic biotransformation and renal excretion, supporting poor free-aglycone bioavailability after sustained oral dosing [57]. These data together explain reports that oral bioavailability of the parent phenols is low and that formulation strategies (e.g., enteric or encapsulation) are used to enhance exposure [57,58].

Preclinical evidence indicates divergent central effects: carvacrol is lipophilic and can reach the central nervous system with neuroprotective actions in some injury models, whereas thymol has been associated with BBB disruption and behavioural toxicity in rodents. Abbasloo et al. (2023) [19] showed that carvacrol, administered intraperitoneally after diffuse traumatic brain injury in rats, was sufficiently lipophilic to diffuse across the BBB. At 200 mg/kg, it reduced BBB permeability, oxidative markers, and metalloproteinase-9 while restoring tight-junction proteins. In contrast, a study in mice reported that 30-day oral thymol (20, 40 mg/kg) induced BBB breakdown, accompanied by memory impairment and neurotoxic changes in mice, indicating that thymol exposure can adversely affect BBB integrity and cognitive outcomes under the tested conditions [59].

Metabolic and safety considerations are linked to both limited free systemic exposure and enzyme-mediated clearance. CYP2A6 has been identified as the predominant human cytochrome P450 isoform catalysing the phase I metabolism of thymol and carvacrol in human liver microsomes, implying a potential for drug–drug interactions involving CYP2A6 substrates or inhibitors [60]. Human data for thymol show predominant elimination as sulphate and glucuronide conjugates rather than the free parent compound following oral dosing [55]. Toxicological reviews note generally low acute toxicity at typical use levels but also report cell-line cytotoxicity and dose-dependent adverse effects in some models, underscoring the need for dose control and further human pharmacokinetic-toxicodynamic correlation studies [61,62].

In view of the poor bioavailability of both compounds, researchers have developed advanced nano-formulations and synthetic structural derivatives. Medhat et al. (2019) [38] evaluated a 24 nm spherical oil-in-water nanoemulsion stabilised with Tween 80 in an AlCl_3_-induced AD model. The nanoemulsion was markedly more effective than bulk carvacrol oil, significantly restoring brain monoamine neurotransmitters and brain SOD, while reducing oxidation products and acetylcholinesterase activity. This superior efficacy was attributed to the nanoemulsion’s enhanced thermodynamic stability and significantly higher BBB permeation. Safarbalou & Abbasi (2024) [39] compared free thymol to soy phosphatidylcholine LET. The lipid bilayer coating protected the volatile monoterpene from systemic clearance and oxidation. Consequently, LET (at 40 and 80 mg/kg) achieved significantly higher passive avoidance step-through latencies, a more pronounced suppression of pro-inflammatory cytokines and greater recovery of hippocampal BDNF compared to equivalent doses of free thymol. To address the relatively weak cholinesterase inhibition of raw monoterpenes, synthetic chemists have successfully modified their structures. Gomes et al. (2026) [40] designed selenium-containing carvacrol hybrids via copper-catalysed click chemistry. The top triazole-linked organoselenium compounds, **18e** and **18h**, exhibited exceptionally potent, nanomolar-range inhibition of acetylcholinesterase, with IC_50_ values of 75 nM and 93 nM, respectively. Kurt et al. (2017) [41] synthesised carbamate-substituted derivatives, showing that several carvacrol carbamate compounds and carvacrol-substituted amide derivatives achieved lower AChE IC_50_ and BuChE IC_50_ than raw compounds. Synthetic carvacrol-derived sulphonamides were reported to significantly reverse STZ-induced cognitive amnesia in mice, restore brain GSH and reduce lipid peroxidation [43].

To translate the neuroprotective potential of carvacrol and thymol into clinical practice, future research must prioritise establishing standardised preclinical baselines, mapping detailed pharmacokinetic profiles, and defining long-term safety parameters. This requires resolving species-specific and duration-dependent discrepancies in metabolic models, such as the divergent body weight and glycaemic outcomes observed in high-fat diet-fed rodents, while validating systemic efficacy across standardised age- and gender-matched cohorts in multi-pathological states. Concurrently, systematic in vivo absorption, clearance, and BBB transport kinetics must be mapped alongside chronic, multi-month toxicology screens to monitor organ-specific accumulation and define safe human dosing margins. Crucially, future combination designs must employ systematic checkerboard assays to map combination index profiles, thereby identifying synergistic ratios while avoiding antagonistic interactions.

Subsequent translational efforts must focus on optimising advanced drug delivery systems, exploiting stereochemical structure-activity relationships (SAR), and initiating human clinical trials guided by robust biomarker-based outcomes. Developing standardised spherical nanoemulsions and liposome-encapsulated formulations is essential to protect monoterpenes from rapid in vivo oxidation, overcome volatility, and maximise central bioavailability. In parallel, future chemical synthesis programs should exploit the isomeric differences between carvacrol’s para-hydroxyl and thymol’s meta-hydroxyl configurations to design targeted scaffolds, including triazole-linked selenium-carvacrol hybrids and carbamate derivatives that elevate AChE inhibition to the nanomolar and low-micromolar ranges. Finally, to transition successfully to randomised human clinical trials, protocols must integrate objective central biomarker outcomes, specifically monitoring central IRS-1/AKT/GSK-3β phosphorylation dynamics, neurogenic and synaptic maturation proteins like doublecortin, reelin, and LRP8, and real-time positron emission tomography to evaluate beta-amyloid deposition and microglial neuroinflammation in vivo.

This scoping review has several methodological and evidence-based limitations that should be considered when interpreting the findings. First, in accordance with standard scoping review methodology, we did not formally perform a critical appraisal or risk-of-bias assessment of the included studies; consequently, studies with suboptimal methodological quality or reporting standards may be present in the synthesis. Second, the search strategy was restricted to English-language publications, meaning language and publication bias cannot be entirely excluded. Third, all primary studies identified in this review relied exclusively on preclinical in vitro, in vivo, or in silico models, and no human clinical trials were available. As a result, direct conclusions regarding clinical efficacy, optimal therapeutic dosing, and human safety profiles cannot yet be drawn.

## 4. Methods

This scoping review was designed according to the framework of Arksey and O’Malley [63] and in compliance with the Preferred Reporting Items for Systematic Reviews and Meta-Analyses extension for Scoping Reviews (Table S1) [64]. The following steps were adopted: (1) identifying the research question; (2) identifying the relevant studies; (3) study selection; (4) charting the data; (5) collating, summarising and reporting the results. The protocol of this scoping review has been registered in the Open Science Framework (URL: https://osf.io/cpy4t/; assessed on 3 September 2026).

### 4.1. Identifying the Research Question

The current scoping review addressed the question: “What are the effects of the carvacrol and thymol on AD?” Based on Population, Intervention, Comparator, and Outcomes (PICO) framework, “Population” refers to in vitro neuronal cell models, animal models of AD and patients diagnosed with AD. For the in vivo studies, classic and non-classic AD models, such as hypertension and diabetes-related cognitive decline models, were considered. Non-classic AD models (e.g., metabolic, neuroinflammatory, or hypoperfusion-induced cognitive impairment) were included because AD is a complex, multifactorial disorder in which vascular dysfunction, neuroinflammation, and metabolic stress act as key pathophysiological drivers alongside classical Aβ and tau pathology. “Intervention” refers to carvacrol or thymol, each monoterpene alone or both monoterpenes in combination. “Comparator” refers to untreated or vehicle-treated negative controls in preclinical studies, or patients receiving placebo or standard therapies. “Outcomes” refer to cognitive performance measures, neuronal viability, and biochemical markers relevant to AD pathology (e.g., Aβ levels, oxidative stress markers, inflammatory mediators, apoptotic markers, cholinesterase activity, and other signalling markers).

### 4.2. Identifying Relevant Studies

A literature search was performed on three electronic databases, i.e., PubMed, Scopus and Web of Science in February 2026, using the search string: (carvacrol OR thymol) AND (dementia OR Alzheimer’s OR “cognitive impairment”) (Table S2). The search string was applied to titles and abstracts to avoid nonspecific results. No other filters were applied. All items from the inception of databases to the date of the search were included. In this search, all primary studies that used in vitro, in vivo, or in silico models to investigate the effects of carvacrol and thymol on AD were considered.

Articles without primary results, such as reviews, perspectives, commentaries, letters to the editor, books and book chapters were not considered. Conference abstracts and proceedings were not included due to incomplete data and potential overlap with the full reports. Articles not written in English were excluded. Studies that used formulations combining carvacrol or thymol with other compounds, without an independent group treated with the monoterpenes, were excluded.

### 4.3. Study Selection

The search results were downloaded from the three electronic databases and organised using a standard Google Sheet (Google Inc., Mountain View, CA, USA). Duplicated items were removed, and the results were manually verified to ensure successful deduplication. The titles and abstracts were independently screened by two authors (S.A.S., K.-Y.C.) using the inclusion and exclusion criteria. Next, the full texts of the eligible items were obtained and screened by the same researchers. Disagreements were resolved by discussion with the third author (J.K.T.).

### 4.4. Charting the Data

The author S.A.S. extracted relevant information from the selected studies, including researchers, publication years, study design (subjects or disease models used, dosage of carvacrol, thymol or both, treatment period), limitations and major findings. This was done using a standard Google Sheet (Google Inc., Mountain View, CA, USA). The extracted data were verified by another author (K.-Y.C.). A discussion was held to resolve any discrepancies found in the extracted data.

### 4.5. Collating, Summarising and Reporting the Results

Statistical data pooling was not conducted because the included studies were heterogeneous in terms of study designs, AD models, outcome measures, treatment durations, monoterpene dosages and reporting formats. The results were summarised descriptively based on the critical pathological features of AD. This includes cognitive behavioural outcomes, cholinergic function, oxidative stress markers, neuroinflammatory cytokine levels and apoptotic pathways. The protective effects of carvacrol and thymol in AD, the mechanisms proposed across the included studies, the current research gaps and the limitations of this review are discussed in the Section 2 and Section 3.

## 5. Conclusions

The available preclinical evidence generally suggests that carvacrol and thymol may exert protective effects across direct AD pathology models, AD-relevant risk models, and general cognitive impairment paradigms. The evidence is consistent with putative multi-target mechanisms, including attenuation of oxidative stress, suppression of neuroinflammatory cytokines, inhibition of cholinesterase activity, and preservation of synaptic signalling pathways. Chemical modifications to the monoterpene scaffold and encapsulation within advanced delivery systems (such as liposomes and nanoemulsions) have the potential to enhance potency and brain exposure relative to native forms. Despite these encouraging preclinical signals, substantial barriers prevent immediate clinical translation. The current literature is restricted entirely to animal and cell models, exhibits high heterogeneity in dosing and treatment durations, and frequently reports non-linear exposure-response relationships. Furthermore, because a formal critical appraisal was not conducted as part of this scoping framework, the methodological certainty and risk of bias across primary studies remain unrated. To bridge this translational gap, future work must prioritise standardised preclinical AD models, comprehensive pharmacokinetic and toxicological evaluations, structure–activity relationship optimisation and carefully designed early-phase human clinical trials incorporating validated biomarker endpoints.

## Figures and Tables

**Table 1:** Summary table for the effects of carvacrol.

Authors (Year)	Study Design	Model Characteristics	Treatment Characteristics	Findings (Carvacrol/Thymol Group vs. Negative Control)		
				Increased	Decreased	Unchanged
Direct AD models						
Medhat et al. (2019) [38]	In vivo	Animals: Forty male albino rats Models: AlCl_3_	Treatment: Carvacrol nanoemulsion Dose: 20 μL/kg body weight (p.o., twice weekly) Negative control: vehicle Positive control: n/a Treatment period: 30 days	Brain SOD activity & GSH levels	Brain neurotransmitter levels (nor-epinephrine, serotonin, and dopamine) Urinary 8-OHdG levels Brain AOPP levels Brain TBARS levels Brain cholinesterase activity Brain expression of COX-1 and COX-2	n/a
Celik Topkara et al. (2022) [28]	In vitro In vivo	In vitro: Cells: SH-SY5Y cells Model: Aβ_1–42_, 20 μM In vivo: Animals: Adult male Wistar rats Model: Bilateral IHP Aβ_1–42_ injection	In vitro: Treatment: Carvacrol (10, 20, 50, and 100 μM) Negative control: 0.1% DMSO Treatment period: 24 h In vivo: Treatment: Carvacrol (25, 50, and 100 mg/kg; i.p.) Negative control: 10% Tween 80, 1% DMSO in saline (i.p., twice daily) Positive control: Galantamine (3 mg/kg) Treatment period: 3 weeks	In vitro: Cell viability (at 10, 20 and 50 μM in SH-SY5Y cells) In vivo: Passive avoidance test: step-through latency (50 and 100 mg/kg) Morris water maze probe trial: Time spent in the target quadrant (50 and 100 mg/kg) SOD & CAT activity, GSH level in the hippocampus (at 50 and 100 mg/kg)	In vitro: Apoptosis of SH-SY5Y cells In vivo: Morris water maze (acquisition): Escape latency and distance travelled (at 50 and 100 mg/kg) MDA levels in the hippocampus (at 50 and 100 mg/kg)	In vivo: Swimming speed in the Morris water maze
Kazemi et al. (2024) [26]	In vivo	Animals: Adult male Wistar rats (200–250 g) Models: i.c.v. injection of Aβ_1–42_)	Treatment: Carvacrol (25, 50 and 100 mg/kg; p.o.) Negative control: Saline (p.o., oral gavage, same schedule as treatment groups) Positive control: n/a Treatment period: 8 weeks	Slope of field excitatory postsynaptic potentials (fEPSP) in the hippocampal CA1 area (50 and 100 mg/kg) Amplitude of fEPSP (50 and 100 mg/kg)	n/a	n/a
AD-associated risk models						
Deng et al. (2013) [32]	In vivo	Animals: Adult male Wistar rats Models: Diabetes induced by a single streptozotocin injection (60 mg/kg)	Treatment: Carvacrol (25, 50, and 100 mg/kg; p.o.) Negative control: 0.5% methylcellulose Positive control: n/a Treatment period: 7 weeks	Morris water maze probe trial: time spent in the target quadrant SOD activity & GSH levels in the cerebral cortex and hippocampus Body weight at the end of the study	Morris water maze (acquisition): Escape latency MDA levels in the cerebral cortex and hippocampus Pro-inflammatory cytokines (TNF-α and IL-1β) in the cerebral cortex and hippocampus NF-κB p65 unit expression (nuclear fraction) in the cerebral cortex and hippocampus Caspase-3 activity in the cerebral cortex and hippocampus Plasma glucose at end of study	n/a
de Souza et al. (2020) [43]	In vivo	Animals: Adult female Swiss mice Model: Streptozotocin (3 mg/kg, i.c.v.)	Treatment: Carvacrol and five synthetic carvacrol-derived sulphonamides (compounds **1**–**5**) (50 mg/kg; p.o.) Negative control: Vehicle (distilled water with 2% Tween 80; i.p.; same schedule) Positive control: 0.5 mg/kg Galantamine Treatment period: 12 days	AChE inhibition (in vitro; carvacrol showed 19.3% inhibition) Inhibitory avoidance task: step-through latency (specifically for carvacrol and compounds **1**, **3** and **5**) GSH levels in hippocampus for compounds **1**, **2**, **3** and **5** open-arm frequency in compound **4**.	TBARS levels in the hippocampus in compounds **1**, **2**, **3** and **5**. Open-arm time permanency in compounds **4** and **5**; open-arm frequency in compounds **4** and **5**.	Open field test: Elevated plus maze: Locomotor activity Inhibitory avoidance test
Raeini et al. (2020) [33]	In vivo	Animals: Adult male Wistar rats Model: chronic cerebral hypoperfusion (CCH) model induced by permanent bilateral occlusion of the common carotid arteries	Treatment: Carvacrol (25 & 50 mg/kg; i.p.) Negative control: normal saline Positive control: n/a Treatment period: 8 weeks	Morris water maze probe trial: Time spent in the target quadrant (50 mg/kg) Number of healthy/viable pyramidal neurons in the hippocampal CA1, CA2, and CA3 regions Neuronal density Hippocampal SOD Hippocampal CAT Percentage inhibition (25 mg/kg only)	Morris water maze (acquisition): escape latency and swimming distance to find the hidden platform MDA levels in the hippocampus Neuronal cell death and morphological damage in the hippocampus	
Bayraktar et al. (2025) [34]	In vivo	Animals: Adult female Sprague-Dawley Model: OVX + two-kidney, one-clip (2K1C) renovascular hypertension surgery	Treatment: Carvacrol (40 mg/kg; p.o.) Negative control: Distilled water Positive control: Perindopril (2 mg/kg) Treatment period: 7 weeks	Morris water maze probe trial: Time spent in the target quadrant Novel object recognition test: Exploration time SOD & CAT activity, GSH-level activity in brain tissue	Systolic blood pressure Morris water maze (acquisition): Escape latency and swimming distance Aβ_1–42_ accumulation in the hippocampus and cortex Brain TNF-α & IL-1β levels Brain MDA levels Brain MPO activity Brain caspase-3 levels	Morris water maze: Swimming speed Open field test: Total distance moved
General cognitive impairment/Unspecific models						
Zengin Kurt et al. (2020) [42]	In vitro In silico	Enzymes: AChE and BuChE In silico models: Molecular docking algorithms and 100 ns molecular dynamics (MD) simulations Permeability model: parallel artificial membrane permeability assay (PAMPA-BBB) to predict BBB penetration Toxicity model: MetaCore/MetaDrug platform for pharmacokinetic and toxicity profile predictions	Compounds: 23 novel carvacrol derivatives containing an amide moiety as a linker The 23 novel derivatives (**5a**–**5w**) all share a common carvacrol-phenoxyacetyl scaffold with an amide linker, with various aliphatic or aromatic amine substituents. Potent compounds: Compound **5v** [2-(5-isopropyl-2-methylphenoxy)-N-(quinolin-8-yl) acetamide] and **5w** Dose: IC_50_ from acute enzyme incubation Negative control: n/a Positive control: galantamine, donepezil, tacrine and rivastigmine	Binding affinity: Strong binding energy and stability in the active sites of AChE and BuChE BBB Permeability: Compound **5v** showed high predicted permeability like standard drugs Structural interactions: Hydrogen bonds and π-π interactions between derivatives and enzyme residues (e.g., compound **5w** with carbonyl oxygen interaction)	AChE activity: Significant inhibition; compound **5v** had an IC50 of 1.93 µM (149-fold more potent than carvacrol alone) BuChE activity: Significant inhibition; compound **5v** had an IC_50_ of 0.05 µM (82-fold more potent than carvacrol alone) Predicted toxicity: Studied molecules showed generally acceptable toxicity profiles in computer-based models	AChE inhibition: Compound **5v**’s IC_50_ (1.93 µM) was statistically similar to the standard drug Galantamine (IC_50_ = 2.21 µM)
Amooheydari et al. (2022) [36]	In vivo	Animals: Adult male Wistar rats (200–250 g) Model: LPS injection (1 mg/kg)	Agents: Carvacrol (25 and 50 mg/kg; i.p. daily) Negative control: 1% Tween 80 dissolved in normal saline Positive control: n/a Treatment period: 2 weeks before and during 5 days of LPS exposure	Morris water maze probe trial: Percentage of time spent in the target quadrant (25 mg/kg) Total thiol concentration in cortex (25 mg/kg)	Morris water maze (acquisition): escape latency (25 mg/kg carvacrol, across all 4 training days) TNF-α levels in hippocampus (25 mg/kg carvacrol) TBARS levels in hippocampus (25 and 50 mg/kg carvacrol) and cortex (25 mg/kg carvacrol)	TNF-α levels in the cortex Total thiol concentration in the hippocampus
Caputo et al. (2023) [44]	In vitro	Cell: Human neuroblastoma SH-SY5Y cells Model: Oxidative stress induced by H_2_O_2_ (150 μM)	Treatment: Carvacrol (1, 10 and 100 μM) Negative control: Vehicle (ethanol < 0.1%) Positive control: Galantamine (for enzyme inhibition assays) Treatment period: 24 h	Cell viability (at 10 and 100 μM) H_2_O_2_ scavenging activity in a cell-free assay	AChE activity (IC_50_ = 60.18 μg/mL) BuChE activity (IC_50_ = 84.82 μg/mL) α-amylase activity (IC_50_ = 70.83 μg/mL) Intracellular ROS levels	n/a
Valentim et al. (2024) [37]	In vivo	Animals: Adult male Swiss mice (25–30 g) Models: Chronic unpredictable mild stress for 4 weeks	Treatment: Carvacrol (50 mg/kg; p.o.) Negative control: distilled water with 5% Tween 80 Positive control: Fluoxetine (10 mg/kg) Treatment period: 4 weeks	Sucrose preference (indicating reduction in anhedonia) Open field test: time spent in the centre Novel object recognition: exploration time of the novel object Rota-rod: Latency GSH levels in the hippocampus and prefrontal cortex	Forced swim test: Immobility time I Tail suspension test: depressive-like behaviour MDA and nitrite levels in the hippocampus and prefrontal cortex IL-1β and TNF-α in the brain	Open field test: Number of crossings
Gomes et al. (2026) [40]	In silico In vitro	Electrophorus electricus AChE enzyme and equine serum BuChE enzyme;	Treatment: Carvacrol and 12 synthetic carvacrol-based organoselenium hybrids (compounds **7a**–**l**) Dose: IC50 derived from enzyme incubation Negative control: n/a Positive control: donepezil and galantamine (for AChE/BChE inhibition); Trolox and gallic acid (for antioxidant assays)	Radical scavenging activity (especially for selenium-containing hybrids) Binding affinity to AChE and BChE active sites Antioxidant activity (electrochemical—cyclic voltammetry): Radical scavenging/antioxidant potential: compounds **18L** (sulfur, 4-Cl-Ph; oxidation energy = 0.96 V), **18f** (selenium, 1,3,5-Me-Ph; 1.095 V), and **18c** (selenium, 4-Me-Ph; 1.123 V) showed the lowest oxidation energies, indicating the strongest antioxidant potential among the series DFT-calculated and experimental oxidation potentials showed a strong linear correlation (R^2^ = 0.90), confirming computational reliability In silico (molecular docking): Binding affinity to human AChE active site (PDB ID: 4M0E): all 12 compounds docked with ChemPLP scores ranging from 82.61 (**18L**) to 98.56 (**18e**), all exceeding the co-crystallised ligand score (78.62) Compound **18e** (naphthyl-Se): highest docking score (98.563); key interactions with TRP286 (π–π stacking, cation–π), TYR341 (hydrophobic and hydrogen bonding), TYR72, TYR124, TYR337, ASP74, PHE295, PHE297, PHE338 Compound **18h** (butyl-Se): second highest docking score (89.617); similar binding mode within the peripheral anionic site (PAS) Strong correlation between docking scores and experimental IC_50_ values: R^2^ = 0.9377	AChE activity (Carvacrol alone showed 22.8% inhibition at 100 μM; hybrids showed higher potency with IC_50_ values as low as 15.6 microM) BuChE activity (hybrids showed significant inhibition, e.g., compound **7i** with IC_50_ of 19.3 μM)	n/a

Abbreviations: 8-OHdG, 8-hydroxyguanosine; 2K1C, two-kidney, one-clip; Aβ, amyloid-beta; AChE, acetylcholinesterase; AD, Alzheimer’s disease; AlCl_3_, aluminium chloride; AOPP, advanced oxidation protein products; BBB, blood–brain barrier; BuChE, butyrylcholinesterase; CA1/CA2/CA3, cornu ammonis regions 1, 2, and 3; CAT, catalase; CCH, chronic cerebral hypoperfusion; COX-1/COX-2, cyclooxygenase-1 and -2; DFT, density functional theory; DMSO, dimethyl sulfoxide; EPM, elevated plus maze; fEPSP, field excitatory postsynaptic potential; GSH, reduced glutathione; H_2_O_2_, hydrogen peroxide; IC_50_, half-maximal inhibitory concentration; i.c.v., intracerebroventricular; IHP, intrahippocampal; IL-1β, interleukin-1 beta; IL-6, interleukin-6; i.p., intraperitoneal; LPS, lipopolysaccharide; LTP, long-term potentiation; MD, molecular dynamics; MDA, malondialdehyde; MPO, myeloperoxidase; n/a, not applicable; NF-κB, nuclear factor kappa B; OVX, ovariectomized; PAMPA-BBB, parallel artificial membrane permeability assay for blood–brain barrier penetration; PAS, peripheral anionic site; p.o., per os (orally); ROS, reactive oxygen species; SH-SY5Y, human neuroblastoma cell line; SOD, superoxide dismutase; TBARS, thiobarbituric acid reactive substances; TNF-α, tumour necrosis factor-alpha.

**Table 2:** Summary table for the effects of thymol.

Authors (Year)	Study Design	Model Characteristics	Treatment Characteristics	Findings (Carvacrol/Thymol Group vs. Negative Control)		
				Increased	Decreased	Unchanged
Direct AD models						
Asadbegi et al. (2017) [46]	In vivo	Animals: Adult male Wistar rats Model: HFD for 8 weeks, followed by bilateral intrahippocampal injection of Aβ_1–42_ (2 μL, 100 μg) into dentate gyrus	Treatment: Thymol (30 mg/kg/day, p.o.) Positive control: n/a Negative control: sunflower oil (vehicle) Treatment period: 4 weeks	Step-through latency (24 and 48 h) Morris water maze probe trial: Time in target quadrant Viable pyramidal neurons (CA1)	Morris water maze (acquisition): Escape latency and travelled distance Time in dark compartment (passive avoidance) Amyloid plaques Serum TG, VLDL, cholesterol, insulin	Serum glucose Body weight, HDL and LDL Acquisition latency and number of trials
Asadbegi et al. (2018) [47]	In vivo electrophysiology	Animal: Adult male Wistar rats Diet: HFD followed by bilateral intrahippocampal injection of Aβ_1–42_ (2 μL, 100 μg) into dentate gyrus	Treatment: Thymol (30 mg/kg/day, p.o. in sunflower oil) Positive control: n/a Negative control: sunflower oil (vehicle) Treatment period: 4 weeks	EPSP slope and population spike amplitude (LTP) Total antioxidant capacity Total thiol groups	Serum MDA Total oxidant status Serum TG, VLDL, cholesterol Amyloid plaques	Serum LDL Body weight
Bitmez et al. (2024) [30]	In vivo	Animal: Adult male Wistar albino rats Model: D-galactose (60 mg/kg/day, i.p.) + AlCl_3_ (200 mg/kg/day, p.o.)	Treatment: Thymol (30 mg/kg/day, p.o.) Positive control: n/a Negative control: vehicle (sunflower oil) Treatment period: 4 weeks	Novel object recognition test: Recognition index and discrimination index (24 h)	Radial arm maze: Working memory errors, reference memory errors, maze time Brain ApoE	Serum and Brain MDA Serum and brain GSH Brain Aβ_1–42_ Brain reelin and LRP8
Foroughi et al. (2024) [29] #	In vitro	Cells: PC12 cells Model: Formaldehyde (0.35 mM, 24 h)	Treatment: Thymol (25 and 35 μg/mL) Other group: Allium cepa (500, 1000 μg/mL) Quercetin (20, 40 μM) Thymus vulgaris (0.3, 0.4 ppm) Positive control: n/a Negative control: not treated	Cell survival Total antioxidant capacity PP2A gene expression	Apoptosis rate GSK-3β gene expression NMDAR gene expression	APP gene expression BACE1 gene expression
Safarbalou & Abbasi (2024) [39]	In vivo	Animals: Male Wistar rats Model: Bilateral intrahippocampal injection of Aβ_1–42_ oligomers (1 μg/μL per site) into CA1 region.	Treatment: Free thymol (40 or 80 mg/kg/day, p.o.) Liposome-encapsulated thymol (LET) (40 or 80 mg/kg/day, p.o.) Positive control: n/a Negative control: not specified Treatment period: 21 days	Shuttle box test: Step-through latency Hippocampal BDNF (LET > free)	Serum and hippocampal IL-1β, IL-6, TNF-α, COX-2 (dose-dependent; LET more effective)	n/a
AD-associated risk models						
Fang et al. (2017) [31]	In vivo	Animals: Male C57BL/6J mice Model: HFD	Treatment: Thymol (20 or 40 mg/kg/day, p.o.) Positive control: Metformin (200 mg/kg/day, p.o.) Negative control: 0.3% carboxymethyl cellulose sodium Treatment period: 12 weeks	Morris water maze probe trial: Time in target quadrant Hippocampal SOD Hippocampal Nrf2 and HO-1 Hippocampal p-AKT (Ser473) and p-GSK-3β (Ser9)	Morris water maze (acquisition): Escape latency Body weight gain Fasting insulin and HOMA-IR Hippocampal Aβ deposition & p-Tau (Ser396) Hippocampal MDA, IL-1β, TNF-α Hippocampal p-IRS-1 (Ser307)	n/a
General cognitive impairment/Unspecific models						
Guo et al. (2022) [48] #	In vivo	Animal: Male ICR mice Model: D-galactose (150 mg/kg/day, s.c.)	Treatment: Thymol (20, 40, or 80 mg/kg, p.o.) Other groups: *Monarda didyma* L. essential oil (EO): 20, 40, or 80 mg/kg Positive control: Donepezil (3 mg/kg, p.o.). Negative controls: Saline Treatment period: 8 weeks	Morris water maze probe trial: increased crossings/time, platform quadrant activity time Step-through test: latency Hippocampal SOD and GPx Hippocampal p-ERK/ERK ratio Hippocampal Nrf2, HO-1, SOD2, NQO-1	Morris water maze (acquisition): Escape latency Hippocampal MDA Hippocampal AChE activity Hippocampal p-P38/P38 ratio Hippocampal neuronal damage	n/a
Hamdan et al. (2022) [45] #	In vivo + docking	Animals: Adult male Dawley rats Model: AlCl_3_ (70 mg/kg/day, i.p.) for 5 weeks.	Treatments: Thymol (30 mg/kg, p.o.) Other treatment groups: Morin (20 mg/kg, p.o.) Thymoquinone (10 mg/kg, p.o.) Combination (COM) of thymol, morin, thymoquinone Physical and mental training (PhM) = forced swimming + Y-maze (2 days/week) COM + PhM (combined) Positive control: n/a Negative controls: Saline Treatment period: 5 weeks	Y-Maze: spontaneous alternation percentage Morris water maze: time spent in target quadrant Brain TAC and SOD Brain neurotransmitter levels (DA, 5-HT, NE) Brain Nrf2 and HO-1 (mRNA & protein) Brain BDNF protein Brain LRP1 protein Brain Wnt3a, β-catenin, p-GSK-3β (inactive) protein	Conditioned avoidance response: no trials to avoid shock Morris water maze (acquisition): Escape latency Brain MDA Brain AChE Brain TLR4 (mRNA & protein) NF-κB, IL-1β and TNF-α (protein) Brain CHI3L1 (protein) BAX/Bcl-2 ratio (mRNA) Brain BACE1, APP, Aβ, p-Tau, ApoE4 protein Brain NLRP3 and caspase-1 (mRNA & protein) Histopathological damage	n/a
Timalsina et al. (2023) [35]	In vivo	Animals: Male ICR mice Model: Scopolamine (1 mg/kg/day, i.p.) Treatment period: 23 days	Treatments: Thymol (50 or 100 mg/kg/day, p.o.) Other group: TASE (*T. ammi* seed extract, 33.7% thymol) (250 or 500 mg/kg/day, p.o.) Treatment period: First 14 days i.p. (pretreatment), then 23 days p.o. Positive controls: Stigmasterol (10 mg/kg) and Donepezil (5 mg/kg). Negative control: Saline	Brain total & free GSH BDNF (~2-fold) p-GSK-3β (Ser9) Morris water maze probe trial: Time spent in the target quadrant, swimming velocity Passive avoidance test: Step-through latency (retention trial) Granule cell layer thickness DCX-positive cells (neurogenesis)	Brain H_2_O_2_, MDA & TNF-α levels Aβ_1–42_ accumulation Morris water maze (acquisition): Escape latency and swimming distance	Brain oxidised GSH
Khan et al. (2023) [49]	In silico + spectroscopy	Target protein: Human transferrin (Tf, PDB ID: 3V83)—679 aa, N- and C-terminal domains with iron-binding pockets. Relevance to AD: Tf regulates free iron, preventing iron-triggered oxidative stress implicated in AD pathogenesis.	Thymol (ligand): Docking: Thymol structure from PubChem (CID: 6989). MD simulation: 300 ns all-atom simulation of Tf–thymol complex. Spectroscopy: Tf (5 mg/mL) titrated with thymol (0–15 μM for UV; 0–7 μM for fluorescence).	Binding affinity (docking −6.1 kcal/mol) UV absorbance (hyperchromism) Fluorescence quenching (K_SV = 1.32 × 10^5^ M^−1^) Slight increases in Rg and SASA Expanded conformational space (PCA/FEL)	Intramolecular H-bonds within Tf (modest reduction)	Overall RMSD (complex stable) Average RMSF (overall flexibility stable) No major structural shifts
Jafni et al. (2025) [50]	In silico + in vitro	Cell line: N2a Neurotoxicity model: Okadaic acid (OA, 100 nM, 24 h)	Treatment: Individual IC_50_ values (AChE inhibition): Vitexin: 135.24 μg/mL (312.9 μM) Thymol: 36.91 μg/mL (245.7 μM) Optimal synergistic combination (CI = 0.53): Vitexin 45.08 μg/mL + Thymol 7.382 μg/mL Positive control: Donepezil (50 μg/mL) Negative control: OA alone Treatment period: Pre-treatment for 2 h, then exposed to OA for 24 h.	Cell viability PP1 gene expression AChE inhibition	MAPK kinases (MEK1/2, ERK1/2, JNK) Other tau kinases (GSK-3β, CAMKII, P70S6K, PAR1)	n/a

Abbreviations: 5-HT, serotonin; Aβ, amyloid-beta; AChE, acetylcholinesterase; AD, Alzheimer’s disease; AlCl_3_, aluminium chloride; APP, amyloid precursor protein; ApoE4, apolipoprotein E4; BACE1, beta-site amyloid precursor protein cleaving enzyme 1; BAX/Bcl-2, pro-apoptotic/anti-apoptotic regulators; BDNF, brain-derived neurotrophic factor; BrdU/NeuN, bromodeoxyuridine/neuronal nuclei (markers of hippocampal neurogenesis); BuChE, butyrylcholinesterase; CA1, cornu ammonis region 1; CAT, catalase; CHI3L1, chitinase-3-like protein 1; CMC-Na, sodium carboxymethylcellulose; COX-2, cyclooxygenase-2; DA, dopamine; DMSO, dimethyl sulfoxide; ERK, extracellular signal-regulated kinase; GPx, glutathione peroxidase; GSH, reduced glutathione; GSK-3β, glycogen synthase kinase-3 beta; HFD, high-fat diet; HO-1, heme oxygenase-1; HOMA-IR, homeostatic model assessment for insulin resistance; i.c.v., intracerebroventricular; IHP, intrahippocampal; IL-1β, interleukin-1 beta; IL-6, interleukin-6; i.p., intraperitoneal; IRS-1, insulin receptor substrate-1; LET, liposome-encapsulated thymol; LRP1, low-density lipoprotein receptor-related protein 1; MD, molecular dynamics; MDA, malondialdehyde; mRNA, messenger ribonucleic acid; n/a, not applicable; NE, norepinephrine; NF-κB, nuclear factor kappa B; NLRP3, NOD-like receptor protein 3; NMDAR, N-methyl-D-aspartate receptor; NQO1, NAD(P)H quinone oxidoreductase 1; Nrf2, nuclear factor erythroid 2-related factor 2; OA, okadaic acid; p.o., per os (orally); PP2A, protein phosphatase 2A; PSD-95, postsynaptic density protein 95; ROS, reactive oxygen species; s.c., subcutaneous; SOD, superoxide dismutase; SOD2, superoxide dismutase 2; TAC, total antioxidant capacity; TASE, Trachyspermum ammi seed extract; TBARS, thiobarbituric acid reactive substances; TLR4, toll-like receptor 4; TNF-α, tumour necrosis factor-alpha; Wnt3a/β-catenin, wingless-related integration site 3a/beta-catenin. Notes: # only the data of the group treated with thymol alone were extracted.

**Table 3:** Summary table for direct comparison between thymol and carvacrol.

Authors (Year)	Study Design	Model Characteristics	Treatment Characteristics	Findings (Carvacrol/Thymol Group vs. Negative Control)		
				Increased	Decreased	Unchanged
Direct AD models						
Azizi et al. (2012) [25] *	In vivo	Animals: Adult male Wistar rats Model: 1. Bilateral IHP injection of Aβ_25–35_ 2. i.p. scopolamine; 1 mg/kg)	Treatment: Carvacrol and thymol (0.5, 1, and 2 mg/kg; i.p.) for 5 days Negative control: Saline or 10% Tween 80 Positive control: n/a Treatment period: 5 days	Both models: Morris water maze: Number of entries into the target quadrant Aβ model: Thymol: 0.5 and 1 mg/kg; carvacrol 1 and 2 mg/kg Scopolamine model: Thymol: 0.5 and 1 mg/kg; carvacrol 1 mg/kg	Both models: Morris water maze: Escape latency during spatial learning acquisition (hidden platform, days 1–4) Aβ model: thymol at 0.5, 1 mg/kg and 2 mg/kg; carvacrol at 1 mg/kg and 2 mg/kg Scopolamine model: thymol and carvacrol at all three doses (0.5, 1, and 2 mg/kg; all *p* < 0.001) Morris water maze: number of entries into the opposite quadrant (both models, same dose ranges as above)	Morris water maze: Swimming speed, Escape latency during the visible platform test
Azizi et al. (2020) [27]	In vitro	Cells: PC12 cells Model: Aβ_25–35_ (10 μM)	Treatment: Carvacrol and thymol (1–100 μM) Negative control: 0.1% DMSO Positive control: Bryostatin-1 Treatment period: 48 h	Cell viability (10–50 μM carvacrol and thymol) PKC activity (10–50 μM carvacrol and thymol)	Intracellular ROS levels (10–50 μM carvacrol and thymol)	n/a
Azizi et al. (2022) [52]	In vivo	Animals: Male albino Wistar rats Model: Aβ_1–42_ (dose not specified; i.c.i.)	Treatment: Carvacrol and thymol (0.5, 1, 2 mg/kg) Negative control: 0.1% Tween 80 Positive control: Bryostatin-1; 0.2 nM Treatment period: initiated one week after surgery, for 5 days of behavioural test	PKC activity and expression (all doses of carvacrol & thymol) PKC protein expression (1–2 mg/kg carvacrol & thymol)	Morris water maze: escape latency (all doses) CA1 pyramidal neurons shrinkage	n/a
Sharma et al. (2023) [51] #	In vitro	In vitro: Cells: human neuroblastoma (SH-SY5Y) cells Model: Aβ_1–42_	Agent used: *Trachyspermum ammi* (Ajwain) extract (0–100 µg/mL) and its bioactives (thymol, p-cymene, and α-terpinene) (µM range) Negative control: 0.1% DMSO Positive control: Galantamine (for AChE inhibition) Treatment period: 24 h	Cell viability of SH-SY5Y cells (50 µg/mL of thymol and carvacrol) Mitochondrial membrane potential (1–25 µg/mL of thymol and carvacrol)	IC_50_ AChE inhibition: thymol 565.7 µg/mL carvacrol 64.21 µg/mL Aβ_1–42_ fibrilization (thymol at 500 µg/mL) Aβ_1–42_ oligomerisation (thymol and carvacrol at 500 µg/mL) Intracellular ROS levels in SH-SY5Y cells (10–50 µg/mL of thymol and carvacrol)	n/a
General cognitive impairment/Unspecific models						
Kaufmann et al. (2011) [53]	In vitro	AChE enzyme from *Electrophorus electricus*	Compounds: Carvacrol and thymol (among other essential oil components) Dose: 0.01 to 10 mM for enzyme incubation Negative control: n/a Positive control: Galantamine	n/a	AChE activity: Carvacrol IC_50_ = 0.21 ± 0.04 mM Thymol IC_50_ > 25 mg/mL (inactive)	n/a

Abbreviations: Aβ, amyloid-beta; AChE, acetylcholinesterase; AD, Alzheimer’s disease; BuChE, butyrylcholinesterase; CA1, cornu ammonis region 1; DMSO, dimethyl sulfoxide; IC_50_, half-maximal inhibitory concentration; i.c.i., intracerebroinfusion; IHP, intrahippocampal; i.p., intraperitoneal; n/a, not applicable; PC12, pheochromocytoma cell line; PKC, protein kinase C; PKCα, protein kinase C alpha; ROS, reactive oxygen species; SH-SY5Y, human neuroblastoma cell line. Notes: # only data of the group treated with monoterpenes are extracted; * the Aβ induction model is a direct AD model, but the scopolamine model is a general cognitive impairment model.

## Data Availability

No new data was created or analyzed in this study. Data sharing is not applicable to this article.

## Supplementary Materials

The following supporting information can be downloaded at: https://www.mdpi.com/article/10.3390/ph19091407/s1, Table S1. Preferred Reporting Items for Systematic reviews and Meta-Analyses extension for Scoping Reviews (PRISMA-ScR) Checklist. Table S2. Complete search string for each database.
