# Supplementary material for: Neuroprotective Effects and Mechanisms of Carvacrol and Thymol in Alzheimer’s Disease: A Scoping Review
Source: Pharmaceuticals (Basel). 2026 Sep 6;19(9):1407. doi: 10.3390/ph19091407 (PMC13610885; doi:10.3390/ph19091407)
Supplement: Supplementary file 1 [file pharmaceuticals-19-01407-s001.zip › Table S2. Complete Search String.pdf]

Table S2. Complete search string for each database

Database	Search string
PubMed	(carvacrol[Title/Abstract] OR thymol[Title/Abstract]) AND (dementia[Title/Abstract] OR Alzheimer's[Title/Abstract] OR "cognitive impairment"[Title/Abstract])
Scopus	(TITLE ((carvacrol OR thymol) AND (dementia OR Alzheimer's OR " cognitive impairment")) OR ABS ((carvacrol OR thymol) AND (dementia OR Alzheimer's OR "cognitive impairment")))
Web of Science	(carvacrol OR thymol) AND (dementia OR Alzheimer's OR "cognitive impairment") (Title) or (carvacrol OR thymol) AND (dementia OR Alzheimer's OR "cognitive impairment") (Abstract)
